# Message in a bottle: Open source technology to track the movement of plastic pollution

**DOI:** 10.1371/journal.pone.0242459

**Published:** 2020-12-02

**Authors:** Emily M. Duncan, Alasdair Davies, Amy Brooks, Gawsia Wahidunnessa Chowdhury, Brendan J. Godley, Jenna Jambeck, Taylor Maddalene, Imogen Napper, Sarah E. Nelms, Craig Rackstraw, Heather Koldewey

**Affiliations:** 1 Centre for Ecology and Conservation, University of Exeter, Penryn, Cornwall, United Kingdom; 2 Zoological Society of London, Regent’s Park, London, United Kingdom; 3 College of Engineering, New Materials Institute, University of Georgia, Athens, Georgia, United States of America; 4 Department of Zoology, University of Dhaka, Dhaka, Bangladesh; 5 WildTeam, Dhaka, Bangladesh; 6 National Geographic Society, Washington, DC, United States of America; 7 International Marine Litter Research Unit, University of Plymouth, Plymouth, United Kingdom; 8 Centre for Circular Economy, University of Exeter, Cornwall, United Kingdom; 9 Icoteq Ltd, Bath, United Kingdom; Universidade de Aveiro, PORTUGAL

## Abstract

Rivers worldwide are now acting as major transport pathways for plastic pollution and discharge large quantities of waste into the ocean. Previous oceanographic modelling and current drifter data have been used to predict the movement and accumulation of plastic pollution in the marine environment, but our understanding of the transport and fate through riparian systems is still largely unknown. Here we undertook a proof of concept study by applying open source tracking technology (both GPS (Global Positing System) cellular networks and satellite technology), which have been successfully used in many animal movement studies, to track the movements of individual plastic litter items (500 ml PET (polyethylene terephthalate) drinks bottles) through the Ganges River system (known as the Ganga in India and the Padma and Meghna in Bangladesh, hereafter known as the Ganges) and the Bay of Bengal. Deployed tags were successfully tracked through the Ganges river system and into the Bay of Bengal marine system. The “bottle tags” were designed and built (e.g. shape, size, buoyancy) to replicate true movement patterns of a plastic bottle. The maximum distance tracked to date is 2845 km over a period of 94 days. We discuss lessons learnt from the development of these plastic litter tags, and outline how the potential widespread use of this open source technology has the ability to significantly increase understanding of the location of accumulation areas and the timing of large inputs of plastic pollution into the aquatic system. Furthermore, “bottle tags” may act as a powerful tool for stimulating social behaviour change, informing science-based policy, and as valuable educational outreach tools for public awareness.

## Introduction

Plastic debris is a complex, persistent pollutant of increasing concern within the environment [1–3]. Plastics make up to 12% of the global waste stream but poor waste governance and its persistence in the environment leads to significant environmental pollution [4]. This is increasingly recognised as a threat to biodiversity, habitat quality, human health and livelihoods [4–13]. A substantial amount of marine plastic debris is thought to originate from inland sources, with rivers acting as major transport pathways; however, currently there are large variations in estimates of riparian inputs of plastic waste to the ocean between studies using modelling [14, 15]. Recent studies estimate that plastic pollution transported via rivers could account for up to 70–80% of plastics present in the marine environment, especially as they are associated with areas of high anthropogenic influence, such as urban centres [1, 16, 17].

The understanding of spatial and temporal patterns and rates of transport via riparian pathways is limited, especially in large catchments [3, 14]. The development of solution-based prevention strategies is reliant on filling this knowledge gap [2]. Historically, understanding of how objects move within the ocean has been garnered from releasing drift cards; for example those released off the South Africa coast between 1964 and 1970 helped inform understanding of circulation rates in the Indian Ocean [18, 19]. More recently, oceanographic modelling and current drifter data have been used to predict the movement and accumulations of plastic pollution from localised coastal areas to a global oceanic scale; integrating information from projects such as The Global Ocean Observing System (GOOS) [20–23]. The distribution of marine debris is thought to be largely governed by surface currents and circulation patterns [18]. Empirical data would greatly complement the existing models, especially those aiming to link inland freshwater inputs into marine systems. Other experimental studies have used artificial streams, observing the movements of replicate plastic bags [24].

Technological advancements in animal tracking have allowed for observations in challenging environments, such as aquatic habitats, where animals are inherently difficult to monitor due to logistical challenges i.e. their dynamic nature and spatial extent [25, 26]. This has allowed movement ecology to rapidly develop in recent years, increasing the understanding of long-term, wide-ranging movements of species, with access to spatio-temporal data crucial for aiding animal conservation [27–32]. The plastic pollution research community can look to animal tracking studies and technology to inform how to approach tracking plastics in aquatic systems, over watersheds and within the ocean.

Recently there have been novel efforts of utilising technology in the tracking of plastic litter. Other projects have focused on ghost gear tracking; the goal of the “GhostNet” was the detection of derelict nets in the Gulf of Alaska [33]. This program (July–August 2003) used numerous examples of technology such as oceanic models, drifter buoys, satellite imagery and remote sensing instruments to locate potential convergence areas where nets were likely to accumulate. Over 100 pieces of individual debris of anthropogenic origin were located using these techniques [33].

The Consortium for Advanced Research on Transport of Hydrocarbon in the Environment (CARTHE) at the University of Miami Rosenstiel School of Marine and Atmospheric Science (RSMAS) released and recorded trackers off the coast of Miami [34]. More recently, an initiative by PAME (Protection of the Artic Marine Environment), “*Plastic in a bottle*” released a trial tracker (2019) offshore of Iceland to simulate how debris moves into and out of Artic waters [35]. Other studies have been working towards satellite imagery to observe areas of floating plastics [36–38]. Applications of novel technology could therefore aid in understanding plastic movements through rivers to marine habitats, including giving insights into the timescale of transit and the quantities that move from inland sources to eventually polluting the open-ocean marine environments [2, 14, 16].

Single-use plastic beverage bottles are now a common and easily identifiable source of marine plastic pollution and make up high proportions of marine litter. In over 25 years (1985 and 2010) of annual coastal clean-ups, plastic beverage bottles were the 5^th^ most reported littered item reported by the International Coastal Cleanup [39] and in 2018, plastic beverage bottles were the 4^th^ most reported item in Bangladesh and the 5^th^ in India during the International Coastal Cleanups [40].

Polyethylene terephthalate (PET) comprises a large majority of the plastic production for the packaging sector and is commonly used for beverage bottles [41]. Baselines are lacking for specific products, however rapid studies of waste composition performed on landfill sites in developing countries found that PET bottles are a large fraction of plastic waste [4, 42]. In this study, modified 500 ml PET bottles were used to house a satellite tag device [43, 44].

### Aims

The Ganges River (known as the Ganga in India and the Padma and Meghna in Bangladesh, hereafter known as the Ganges) is one of the largest river systems in the world with the surrounding basin having a population of several hundred million people. It holds enormous cultural, religious and industrial significance [16]. Rapid population growth in the Ganges basin has resulted in wide-spread agricultural development, urbanisation and industrialisation along the river [45]. India and Bangladesh are rapidly developing countries and the use of plastic materials is increasing exponentially [2, 4]. Recent estimates of global riverine plastic emissions consider the Ganges the second largest contributing river to ocean plastic pollution (after the Yangtze River) with a computed input of 0.12 (range 0.10–0.17) million tonnes per year [15]. However, field data are limited and an understanding of the movement of plastic pollution through the Ganges is a major knowledge gap [16, 46]. During the National Geographic Sea to Source Ganges Expedition (hereafter the Sea to Source Expedition), we undertook a proof of concept study by applying open source tracking technology (previously used on marine vertebrates) aiming to; 1) Use GPS and satellite technology to track and map the movement of plastic litter items (bottles) through aquatic systems; 2) Develop an understanding of the fine-scale movement of plastic pollution through riverine and marine systems, including differences between movement and displacement; 3) Document lessons learnt from applying open source tracking technology to plastic pollution.

## Methods

### Study area

**Fig 1:**
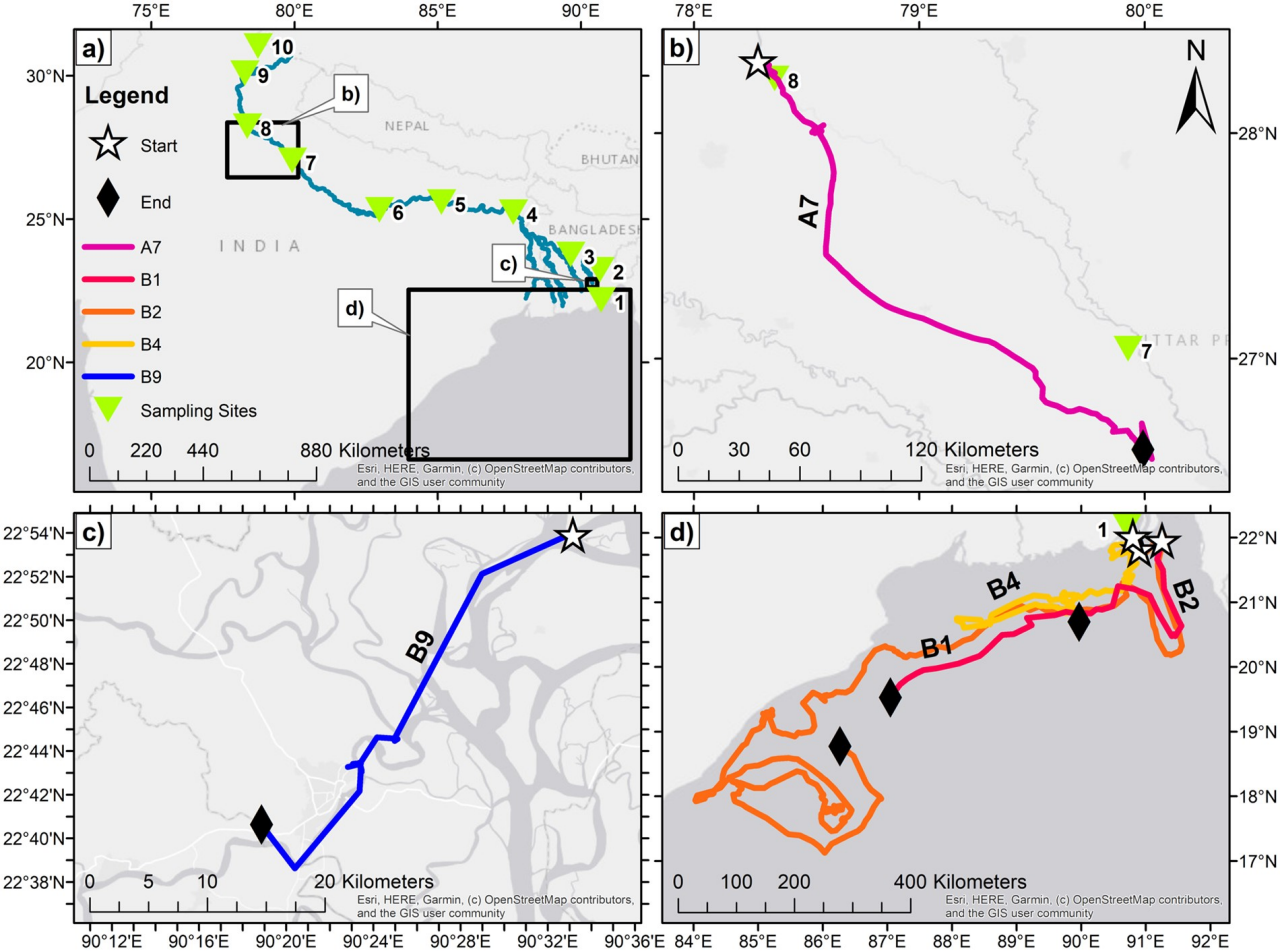
Maps of bottle tag movements a) Expedition site map of the 10 sites in the Sea to Source Expedition; the first three sites in Bangladesh ((1) Bhola, (2) Chandpur and (3) Rajbari) and 7 remaining in India ((4) Sahibganj, (5) Patna, (6) Varanasi, (7) Kannauj, (8) Anupshahar, (9) Rishikesh, (10) Harsil) b) Phase A bottle A7 (pink) deployed in (8) Anupshahar c) Phase B bottle B9 (blue) deployed in (2) Chandpur d) Phase B bottle tags deployed at sea in the Bay of Bengal (B1 (red), B2 (orange)) and (1) Bhola (B4 (yellow)). Star = deployment location, Black diamond = end location, Green triangle = expedition site location. Maps throughout this research article were created using ArcGIS^®^ software by Esri. ArcGIS^®^ and ArcMap^™^ are the intellectual property of Esri and are used herein under license. Copyright © Esri. All rights reserved. For more information about Esri^®^ software, please visit www.esri.com. Contains information from OpenStreetMap and OpenStreetMap Foundation, which is made available under the Open Database License.

The Ganges is the world’s third largest freshwater outlet to the ocean; exceeded only by the Amazon and the Congo river systems [47]. The Ganges originates in the Himalayas at the end of the Gangotri Glacier in the Uttarkashi district of Uttarakhand where it is known as the Bhagirathi River. Once this joins the Alaknanda River in the valleys below, it becomes the Ganges. Further tributaries and rainfall feed the main channel, which flows southeast through the mountainous areas and floodplains of northern India (Fig 1). Numerous urban settlements are located along the river; major population centres include the cities of Kanpur, Varanasi and Patna. The total population within the basin is estimated to be over 474 million [48]. The main channel of the Ganges enters Bangladesh through the Chapai Nawagbganj district where it joins the Brahmaputra and Meghna Rivers and eventually empties into the Bay of Bengal [45, 49]. The Ganges is highly influenced by monsoon weather patterns which result in high precipitation from July to October and tropical cyclones that originate in the Bay of Bengal. The delta region, especially, experiences strong cyclonic storms, both before and after the monsoon season [50]. Due to these weather patterns, the Ganges river basin often experiences flooding, sometimes with severe impacts [51].

### Custom design bottle tags to achieve close to “real world” plastic item

To replicate the flow and movement of a standard 500 ml plastic water bottle in open water (end floatation design based on a half full 500 ml bottle with 50% of the bottle below the water line), it was necessary to design and develop custom electronics and an enclosure that would fit inside the bottle itself, retaining the bottle’s original shape and size, while allowing electronics and batteries to be inserted inside. To achieve this, a computer aided design (CAD) model was built suitable for Computer Numerical Control (CNC) milling, a process where rotary cutting tools are utilized to remove material from a stock unit. The shape and profile were matched to a “real-life” plastic bottle, forming a lid and base that would be sealed using self-tapping screws and a rubber O-ring to protect the electronics inside from water ingress.

**Fig 2:**
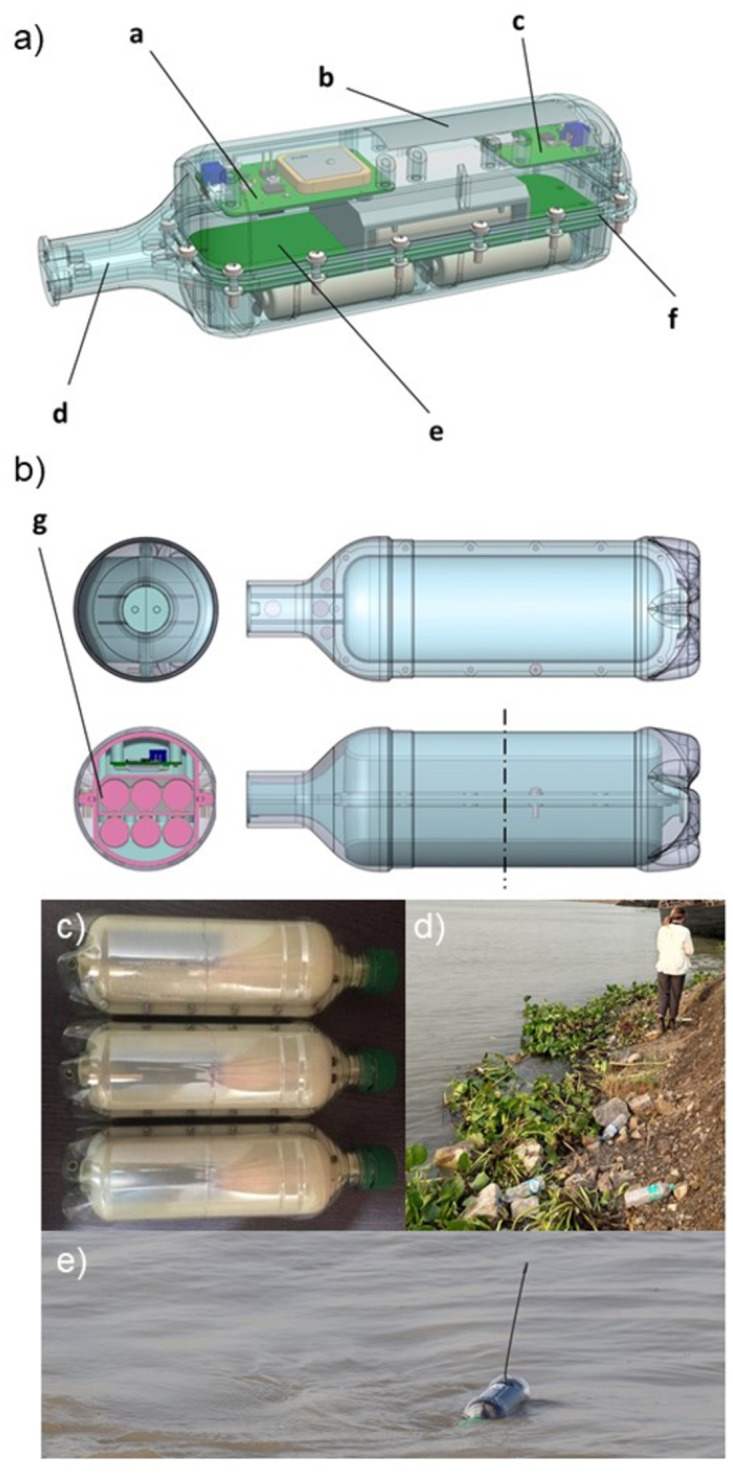
The shape and profile of the bottle tag. a) A transparent schematic showing the seating of batteries and placement of electronics inside the bottle’s enclosure; a = Horizon GPS board, b = Cellular antenna, c = Cellular or Argos satellite board, d = CNC-milled enclosure, e = Battery board, f = O-ring seal. b) Transparent top, side, bottom view and orientation of batteries inside the bottle’s internal cavity; g = Positioning of AA lithium batteries c) Phase A GSM bottle tags d) observed PET bottles in river bank Ganges River e) Phase B satellite bottle tag after deployment.

To factor in the use of regionally appropriate branded plastic bottles manufactured and sold throughout the Ganges catchment, 500 ml plastic bottles were reclaimed locally during an initial scoping trip. In cases where plastic was released for data collection, a system where a subsequent clean-up that removed more plastic than was deployed was implemented during the Sea to Source Expedition. The bottles had bespoke aesthetic curvature to distinguish the individual brands, particularly on the bottle neck, restricting space inside to seat a cylindrical payload. We selected the bottle shape with standard straight wall design and adequate internal cavity space to fit our electrical payload and batteries (Fig 2a).

A two-part internal insert was designed to fit inside a reclaimed 500 ml plastic water bottle, with the addition of an aperture for an ARGOS satellite transmitter on the lid to allow for a 15 cm 1/4 wave antenna to protrude, sealed using a 2-part Araldite epoxy. The cellular antenna was flexible and internal. The lid and base were then screwed together to secure and seal the electronics and batteries inside. We selected a recyclable Acrylonitrile Butadiene Styrene (ABS) thermoplastic as the material and opted for a black / blue finish to conceal the bottle when on open water (S1a Fig). The base of the bottle was then cut to allow the internal enclosure to be inserted inside. The insert’s neck terminated within the water bottle’s screw cap area and a small circular disk was screwed into the bottle cap and then screwed into the insert’s neck to allow the original bottle lid to be connected (S1b Fig).

The internal cavity of the enclosure contained 11 mounting holes to seat an Arribada Horizon GPS printed circuit board (PCB), an Arribada ARGOS ARTIC R2 transmitter and a battery board (https://arribada.org/). Since the bottle itself was cut, and therefore not watertight, we had to ensure the water could move freely around that internal space to maintain balance (S1c Fig). Two larger holes on the neck of the bottle were inserted to help prevent water being trapped in certain sections of the bottle and thereby causing it to float at an angle. The Horizon tracker was installed with the ceramic GPS antenna pointing upwards. A picoblade connector allowed the ARGOS transmitter to be interfaced with the Horizon board using a cable assembly. Next, the ARGOS transmitter was installed under the antenna aperture, with the antenna connected via antennae U.F.L. connector on the ARGOS board and fed upwards into an antenna tube that was sealed using Araldyte epoxy. The cellular tags we deployed switched the ARGOS transmitter PCB for an Arribada Horizon cellular transmitter. The cellular installation process remained the same (S1d Fig). A cable assembly was plugged in to connect the two boards and power supplied by plugging the battery board JST connector into the Horizon board. Power was provided to the ARGOS transmitter via the cable assembly (S1e Fig). Two aluminium strips were then attached to the Printed Circuit Board mount points on each board and screwed in to connect the two Printed Circuit Boards. This extended the ground plane of the antenna and enhanced performance (S1f Fig).

The base of the internal enclosure incorporated an O-ring seal and groove to accept a rubber 2.5mm O-ring. The groove was 1.87mm deep, allowing 75% of the O-ring to be seated and 25% compressed by the flat surface of the lid that was screwed on to seal the unit. Marine-grade lubricant was added to the O-ring before placement to seat it correctly within the groove (S1g Fig). An AA battery holder Printed Circuit Board was then fitted, capable of seating six batteries (base) and three batteries (top), with each battery supported by a groove to hold them securely in place, preventing movement after deployment. A complete unit could therefore hold nine AA batteries in total (S1h Fig). The battery board was screwed into the four mounting holes in the base and six AA lithium batteries installed under the board (S1i Fig).

Once the O-ring seal was installed and seated, the lid was aligned and screwed into place using self-tapping screws. As the lid was sealed, the O-ring was continuously assessed to ensure it remained in the groove and was flush with the lid and base. We inspected the front, rear and sides of the enclosure to ensure that all of the screws were tight. Once sealed, Araldite epoxy was added to the base of the antenna aperture ready for the unit to be inserted inside the plastic bottle and the plastic bottle’s base re-attached (S2 Fig).

After the insertion of the internal enclosure, the plastic bottle’s base was replaced and screwed on to the base of the internal insert to secure it in place and to retain the original shape and profile of the water bottle. A waterproof sticker was appended on the internal insert’s lid with the tag’s identification code and contact information if found to ready the bottles for deployment (S1j Fig).

To successfully receive a GPS fix, the GPS ceramic antenna must be above the waterline and exposed to the sky. In addition, the ARGOS satellite antenna must also be above water and orientated upright, so as close to 90 degrees as possible for optimum results. The design of the bottle took this into account by locating the GPS antenna on the roof of the internal enclosure, with a 15 mm gap between the enclosure wall and Printed Circuit Board. To keep the bottle upright when floating, care was taken to weight the bottle correctly and ensure the centre of gravity would keep the bottle upright when exposed to waves or if capsized. To achieve this, we placed the batteries as low down as possible and spaced them in sets of three within the centre of the bottle. When tested, the bottle successfully righted itself when forcibly capsized, re-positioning the antenna as required if it were immersed by a wave or temporarily submerged (S3 Fig).

To ensure that bottles retained their buoyancy after the addition of electronics and batteries, a programme of work was undertaken to test that the positioning of batteries within the bottle’s cavity achieved the desired centre of gravity and that the increase in weight did not affect the flow of bottles when in the water. To test each bottle, a water tank was used to float and validate various bottle designs until the positioning of the batteries centred the bottle’s gravity and positioned both the cellular and satellite antennas correctly. Modifications were made to the CNC-milled enclosure as necessary and results validated in the test tank.

As it was imperative that the cellular antenna remained above the surface of the water post-deployment for successful transmission, care was taken to weight the bottles. We discovered that the optimum battery capacity was six AA lithium batteries. Each battery added 15 g in weight, resulting in a total weight of 130 g with the addition of the electronics which was sufficient to keep the antenna above the water’s surface but avoid additional drag. When loaded with a maximum capacity of batteries (nine AA Lithium batteries) we found that the additional 45 grams in weight lowered the bottle too much, creating drag and potentially covering the cellular antenna, hence selecting six batteries for the deployment. Two bottle tag configurations were deployed in two different phases (A and B, detailed in Deployments section below). Phase A bottles (GSM tags) were configured to wake every 10,800 seconds (3 hours) and acquire a GPS fix. Each bottle would try to acquire a fix for a maximum of 30 seconds. If successful, the GPS location would be encoded, and a cellular connection made to Amazon Web Services where the data were stored and processed. Phase A bottles had a high idle power consumption (9mA) in comparison to Phase B and so an estimated total life of eight weeks. A second engineering process reduced that to 38uA (9+ years in idle with six AA batteries) for the Phase B deployment.

Phase B bottles were configured to wake up every four hours and would spend up to 30 seconds acquiring a GPS position before returning to a resting state until a satellite passed over. Using a satellite pass prediction algorithm, each bottle would wake and transmit the last GPS location received and a battery status whenever a passing satellite was available with a minimum angle elevation of 40 degrees to maximise the chance of a successful transmission. Data received were then downloaded from the Argos Web Services platform. To keep a constant time, the bottles were programmed to synchronise their internal clocks each time a GPS fix was obtained.

### Deployments

On the Sea to Source Expedition 2019 (which consisted of 10 sampling sites along the length of the Ganges) Phase A (pre-monsoon; May-July 2020) ten GSM (Global System for Mobile) bottle tags were deployed in India; two in Varanasi (site 6), four in Kannauj (site 7), one in Anupshahar (site 8), two in Rishikesh (site 9) and one in Harsil (site 10) (Table 1; Fig 1). Fifteen satellite bottles were deployed in Bangladesh during Phase B (post-monsoon; October-December 2020); four in Bhola (site 1), three in Chandpur (site 2), four in Rajbari (site 3), one in between site 2–3. Three further bottles were released at sea (Bay of Bengal: B1 21.665850° 91.172595°; B2 21.595981°; B3 21.913120° 90.777901°) to better understand the movements of plastic litter items once they enter the marine environment (Table 1; Fig 1). A number of permits were obtained to conduct this work these included; Bangladesh National Government Approval, India National Government Approval, Bangladesh District Approval, India District Approval. In addition, The University of Dhaka & National Geographic Society (NGS) signed a Memorandum of Understanding (MOU) agreement (effective April 30, 2019 expiring June 30, 2020) and the Wildlife Institute of India signed an MOU (effective March 6, 2019 expiring August 1, 2020). These agreements cover all research activities to be conducted, including the ones detailed in this study.

**Table 1:** Tracking information.

No.	Site	Date	Duration (d)	Track (km)	D (km)	D rate (km/day)	Fate
**A1**	6	11/06/19	6	8.8	0.8	0.1	FBP
**A2**	6	11/06/19	4	2.8	1.1	0.3	FBP
**A3**	7	12/06/19	34	36.9	18.2	0.5	UNK
**A4**	7	28/06/19	1	0.8	0.3	NA	NCC
**A5**	7	12/06/19	31	110	20.8	0.7	UNK
**A6**	7	17/06/19	25	133.1	0.2	0	UNK
**A7**	8	17/06/19	51	610	255.3	5	UNK
**A8**	9	21/06/19	24	167	5.6	0.2	FBP
**A9**	9	21/06/19	5	8.7	6.2	1.2	UNK
**A10**	10	25/06/19	NA	NA	NA	NA	NCC
**B1**	Sea	31/10/19	25	780	514.1	20.6	STR
**B2**	Sea	31/10/19	94	2845	590.3	6.3	UNK
**B3**	Sea	31/10/19	6	10.2	7.7	1.3	UNK
**B4**	1	31/10/19	45	941	133.9	3	UNK
**B5**	1	30/10/19	1	28.6	28.6	NA	UNK
**B6**	1	30/10/19	3	4.6	0.52	0.2	UNK
**B7**	1	30/10/19	93	31.7	21.2	0.2	STR
**B8**	2	01/11/19	1	45.1	39.3	NA	UNK
**B9**	2	04/11/19	43	55.7	34.7	0.8	UNK
**B10**	2	02/11/19	21	54.6	47.3	2.3	UNK
**B11**	2 & 3	04/11/19	4	0.5	0.2	0.1	WIN
**B12**	3	08/11/19	22	19.2	7.7	0.4	UNK
**B13**	3	08/11/19	1	0.3	0.0	NA	ADA
**B14**	3	06/11/19	NA	NA	NA	NA	ADA
**B15**	3	07/11/19	NA	NA	NA	NA	ADA

Phase A tags (n = 10) and Phase B tags (n = 15). Tag number, site = expedition site number, duration = number of days tracked, release date, track = total track length (km), D = Displacement (km), D rate = Displacement per day, Fate; release sites, numbers of days tracked and total track length (km). Fate; FBP = found by public, NCC = no cellular connectivity, WIN = Water Ingress, ADA = Antenna Damage, STR = Still transmitted, UNK = Unknown.

### Track reconstruction

Phase A data collection ceased on 07/08/2019. The last downloaded data for Phase B tags were on 02/02/2020 in order to begin analysis and mapping of results of data. All data analyses for track length construction was performed in R 3.6.2 (packages “*sf*”) [52]. The geospatial data recorded for the tags were organised and quality checked in Excel and the location coordinates imported into ArcMap 10.7. Points for each tag location were generated and displayed in WGS GCS 1984 coordinate system. Finally, tag paths shown in Fig 1 were created using the Points to Line tool supplied as part of the ArcMap 10.7 package.

## Results

### Phase A deployments

**Fig 3:**
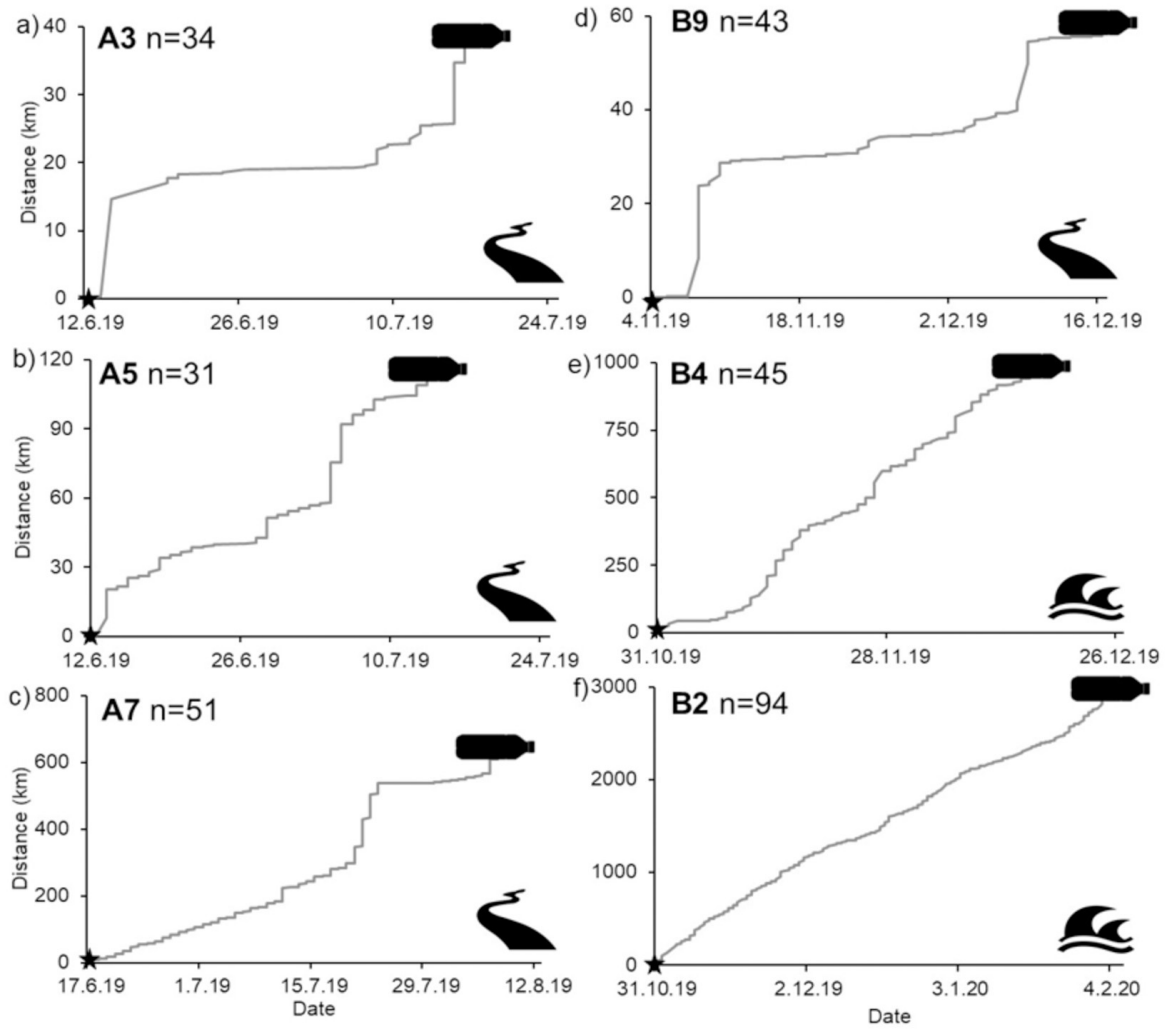
Displacement plots; bottle straight distance displacement movements over time tracked. Star = release. Bottle = tracking finished. a), b), c) & d) river deployments and e) & f) at sea deployments.

Phase A GSM bottle tags (n = 10) deployed in pre-monsoon season were tracked for an average of 20.1 ± 5.7 (mean ± SE; range 1–51) days (n = 9; bottle A10 had no communication after deployment) (Table 1; Fig 1). Total track length obtained was on average 119.5 ± 64.7 km (mean ± SE; range 0.8–609.9 km) (n = 9). These tags had an average displacement of 34.3 ± 27.7 km (mean ± SE; range 0.2–255.3 km) (n = 9) and mean displacement rate of 1.0 ± 0.6 km/day (mean ± SE; range 0.00–5.0 km/day) (n = 8; A4 only tracked for 1 day); all Phase A tags displayed a “stepwise” displacement over the period of tracking (Fig 3). The longest track was from bottle A7 with total length of 609.9 km. This tag was released in site 8 (Anupshahar) and travelled down the main Ganges channel before moving into a constructed side channel, eventually becoming entrapped in a weir or dam (Table 1). A number of the Phase A tags, such as A5, initially moved down the river but then transmitted repeatedly from locations very close together suggesting they were entrapped. Tag A8 was known to have been found shortly after deployment as the SIM card was removed and used in a mobile phone.

### Phase B deployments

#### River

Phase B satellite bottle tags (n = 12) deployed in the river in the post-monsoon season were tracked for an average of 23.1 ± 9.3 (mean ± SE; range 0–92) days (n = 10; B14 and B15 which had no communication once deployed) (Table 1; Fig 1). The total track length obtained was on average 111.9 ± 92.3 km (mean ± SE; range 0.3–941.3 km) (n = 10). These tags had an average displacement 25.4 ± 12.9 km (mean ± SE; range 0.02–133.9 km) (n = 10) and a mean displacement rate of 0.7 ± 0.4 (km/day) (mean ± SE; range 0.0–2.9 km/day) (n = 7). Similarly to Phase A bottles, Phase B river deployed bottles displayed a “stepwise” displacement over the period of tracking (Fig 3). Tags deployed further up the river (site 2 & 3) travelled at varying distances in differing areas of the delta; for example tag B9 travelled down one of the tributaries of the main channel and was tracked for a period 43 days travelling 55.7km before ceasing to transmit for number of possible reasons described below.

#### At sea

Three Phase B bottles were released at sea (Bay of Bengal) to capture the movements of plastic items once they enter the marine environment. These were tracked for an average of 41.6 ± 26.7 (mean ± SE; range 6–94) days (n = 3), with the total track length obtained on average 1,211.7 ± 846.2 km (mean ± SE; range 10.2–2844.6 km) (n = 3). These tags had the largest average displacement 370.7 ± 182.8 km (mean ± SE; range 7.7–590.3 km) (n = 3) and a mean displacement rate of 5.8 ± 9.4 (km/day) (mean ± SE; range 1.3–20.6 km/day) (n = 3). Unlike Phase A and Phase B river-deployed bottles, these bottle tags displayed a continual, consistent displacement over the period of tracking (Fig 3). B2 recorded the longest track of 2,844.6 km (B2) and was tracked for the longest time- period of 94 days; this tag travelled in a westward direction close to the east Indian coastline (Table 1; Fig 1). Tags showed signs of possible water ingress, possibly due to the epoxy solution used to stop the antenna aperture degrading over time. Corrections have been made to address this in preparation for future deployments.

## Discussion

Satellite-tracking technologies are well suited to track small plastic objects without compromising their profile, primarily due to the low-weight and small footprint of transmitters. In this study, we successfully developed a proof of concept method using open source tracking technology to help understand the transport of plastic waste through aquatic systems (both riverine and marine). This work demonstrates a key step forward by moving from observations to tracking movement in “real time” [24]. The bottle tags were carefully designed and built (e.g. shape, size, buoyancy) to replicate true movement patterns of plastic items.

In the South Asian region, 75.3% of waste generated is disposed of via open dumping, following by 15.7% going to sanitary landfills [4]. This means that plastic items (e.g., beverage bottles) are frequently found on the ground or in informal dumping sites as mismanaged plastic waste and make their way into aquatic systems. In coastal fishing communities in India, once plastic bottles are no longer functional (for reuse) they are often discarded on the beaches [53]. Here we see that individual plastic items discarded in this way have the potential to travel large distances (+1,000 km) and move from riverine to open ocean marine systems. Indeed one of PAME’s “Plastic in a Bottle” was recently recovered in Scotland after being tracked across the whole North Atlantic basin (approximately 7,000km over 207 days) [35]. The development of this method offers potential in understanding movements of plastic, as well as locations of potential accumulation, as plastic debris has the ability to persist in the environment for extremely long periods [44]. In a recent study of plastic bottles and containers sampled in the South Atlantic, some were made in the 1970s [44].

The bottle tags deployed in this study displayed differing movement patterns depending on their site of deployment, with 40% of all bottles appearing to become trapped or beached on the riverbank after deployment (after traveling an average of 72.2 km). This observation particularly occurred in Phase A GSM bottle tags in the pre-monsoon season, when water levels were lowest and flows further reduced by damming and abstraction for agriculture [50, 54]. Inter-seasonal riverbank erosion and deposition dynamics play an important role in the movement of plastic waste in riverine systems. Between the pre- and post-monsoon seasons there are significant differences in the water level in the whole river system and river bank height/width, especially in places of relative lower elevation, due to the climatic (sub-tropical monsoon) condition e.g. rainfall [55]. However, many of our tags did not transmit for long enough (reasons discussed below) to monitor large movements in the heaviest periods of the monsoon rain and largest influxes of water into the river. This will be an important consideration in future deployment of such technology.

The three satellite bottle tags deployed at sea all took similar courses once they entered the Bay of Bengal; moving in a westward direction close to the East Indian coastline. The continental shelf in the head of the Bay of Bengal is approximately 160 km wide and narrows to the south [56]. The East India Coastal Current (EICC) is the seasonally reversing western-boundary current of the Bay of Bengal; it flows poleward during February-August and flows equatorward during October- December [57]. The direction of the EICC changes along the coast in January and September, which may explain the movement of B2 that showed an initial equatorward movement before switching to a poleward flow. After this, the bottle potentially entered a cross-shore current and an eddy system, which are also prominent features of the oceanography of the Bay of Bengal region [58]. Taken together, our initial results highlight the potential for this technology to become part of an integrative approach to oceanographic modelling of plastic debris movements in marine systems [21, 59].

The adoption of open source hardware and software allows others to freely replicate, modify, or enhance an existing design by openly sharing the schematics, source code and bill of materials (BOM) necessary to manufacture and repurpose an existing solution. Although leveraging open hardware to drive forward conservation action has been used to great effort across multiple disciplines [60], the publication of open source satellite-based tracking hardware has been limited to date, with the first open source ARGOS transmitter reference design only published in Dec 2019 [61]. Additionally, to negate risk, tried and tested commercial off-the-shelf solutions are often selected over emerging open source solutions that have not yet reached maturity, or have not yet been deployed and used for extended periods of time, limiting uptake [62]. By utilizing the open source Arribada Horizon biologging hardware platform (https://arribada.org), we were able to incorporate Arribada’s low-cost, open source Horizon hardware platform, utilising an Arribada GPS logger, Arribada Cellular Modem and ARGOS transmitter reference design to create the custom bottle tag.

In the future, leveraging both satellite and cellular modems within a single tracker could also enable the use of terrestrial wireless networks to track objects within urban environments where cellular connectivity is available. Dual connectivity helps to reduce overall data costs, as cellular data are cheaper than satellite data and allow for more data to be transmitted from a device, due to the availability of bandwidth in comparison to satellite payloads. By leveraging the ARGOS satellite constellation’s global coverage and roaming cellular connectivity where available, it is possible to monitor the movement of plastic pollution over vast spatial scales, encompassing the open ocean and multiple countries and negating transboundary issues [25, 63], which is essential for issues such as plastic pollution. However, cost remains a major limitation of tracking technology, hence the movement towards lower cost, open source software [60, 64, 65].

### Lessons learnt from deployment

Due to the nature of this proof of concept study, many lessons were learnt from the deployment of both Phase A and B tags.

#### Phase A

*Cellular connectivity*. Phase A GSM bottles tags were released in India, using cellular phone networks to transmit information on location of each bottle obtained via the on-board GPS receiver. Instantaneous GPS cell phone telemetry has been used to record movement data of raccoon dogs (*Nyctereutes procyonoides*) in Japan, allowing description of their habitat use in cities and revealing various spatial and temporal behavioural patterns [66, 67]. However, these studies took place in highly urbanised areas where cellular phone signal remains strong and reliable over the study area, therefore yielding consistent data. A major limitation occurs when countries that have not adopted a GSM system for their mobile network; even so, data recovery is limited to the immediate neighbourhood of communication towers [66, 68]. The reliability of receiving data from the Phase A tags in India was highly variable, primarily due to certain networks providing poor coverage. The best performing bottle tag was switched to use a different cellular network, which resulted in far greater transmissions due to improved network coverage. When the tags were not in urban or suburban areas with dense mobile phone coverage, reception of location data was poor. Therefore, future studies should consider the use of roaming SIMs able to connect to multiple cellular providers and not rely on a single provider regardless of their stated coverage. However, an advantage of GSM tags compared with satellite tags is that there is a much lower cost for ongoing data collection. Furthermore, the use of GSM tags may be a necessity if there are restrictions forbidding satellite transmitters in certain areas or countries.

*Human interference*. For bottle tags that were deployed in highly urbanised areas, there was a particular issue of them being removed from the river system due to human interaction (e.g., a SIM used in bottle tag was found to be used in a mobile device to log into a social media platform). This may be due to their shape, durability and inherent value for reuse or recycling. For instance, at Panambur Beach in Mangaluru, India, PET bottles are reused as floats for fishing nets [53]. PET bottles are a common item captured in the waste stream by the informal waste sector, with one of the highest resale value for waste plastics as opposed to less valuable plastics like straws or plastics bags [69]. Collected PET bottles are typically bailed, sold, reprocessed or traded for recycling; PET and PP (polypropylene) are the most commonly traded scrap plastic with 131 million metric tonnes having been traded between 1988 and 2016 [70]. It is likely that the bottle tags were seen by the public and collected for their value, therefore a less conspicuous design was employed for Phase B.

#### Phase B

*Fisheries pressure*. A major issue arose following the deployment of tags during periods of high fishing pressure; tags became entangled and trapped in active fishing gear, and tags deployed within the river had smaller track lengths and a higher likelihood of discovery and human interference. Bangladesh has a total inland water area of 4.3 million ha. of which 94% is used as open water capture fisheries. These resources play a significant role in the economy and culture, with multiple different types of craft, gears and traps used [71]. Maximum catches (for many of the main composites of the fishery) are obtained during the months from July to December [72]. This means during the period of deployment (October–November) the density of fishers and gear in the river was very high. A 22-day ban had been brought in from 9^th^ October 2019 on fishing in significant spawning events during peak breeding periods for the main commercial fishery for hilsa (*Tenualosa ilisha*) [73]. Unfortunately, the end of the ban coincided with the deployment of the tags in more inland locations resulting in high interactions with fisheries, which resulted in a loss of those tags through collection or destruction.

*Severe weather*. During the period immediately after deployment of the Phase B tags, a severe cyclonic storm (Bulbul) made landfall in Bangladesh on 9^th^ November 2019. The maximum sustained wind speed of 100 kph rising to 120 kph in gusts/squalls and coastal areas received heavy rainfall prior to the cyclone arrival which continued as the cyclone passed [74]. This extreme weather condition could have caused damaged to the tag antennae, which may explain the loss of communication during this period.

*Mechanical issues*. A number of the Phase B tags lost transmission soon after deployment, despite transmitting prior to deployment during self-tests. This indicates an event occurred after deployment that affected transmission. It is possible that these issues are mechanical in certain situations, perhaps due to interactions with fisheries and severe weather. It is possible that the tags were then susceptible to water ingress, perhaps due to issues with the antenna or O-ring seal. For example, tag B10 showed high success with communication but had been stranded in a reed bed for several months so was sheltered. However, the majority of bottles released into open water appeared to stop transmitting for durations of between a few days and over two months. Software issues are unlikely as tags that were constructed and activated at the same time / dates, then stored for future deployments, were still actively transmitting when brought out of storage. This indicates that a mechanical issue post deployment affected tags. To remedy this moving forward, the encapsulation of the bottle’s cavity will prevent any degradation of the O-ring seal or epoxy solution, improving upon the current mechanical design and strengthening bottle tags further.

### Future research

The scope for the use of satellite technology has a significant ability to increase our knowledge of plastic debris movements. Electronic devices are constantly evolving; becoming increasingly miniaturised, more affordable and able to provide even more detailed information on movements in space and time [25]. With smaller devices, it will be possible to track smaller, more lightweight litter items, which are commonly seen polluting both the freshwater and marine environment. Modern tags are also capable of being built with additional sensors that can document the ambient conditions, therefore it might be possible to remotely monitor metrics like water quality in dense areas of plastic debris accumulations [75]. However, the possibility of tracking more plastic litter items comes with ethical considerations about releasing more plastic items into the environment.

Tracking technology in conjunction with mapping tools has increased knowledge of habitat associations for many species of fauna [63]. For plastic pollution, this could be very useful in identifying habitats associated with accumulations of plastic debris and therefore those that are most vulnerable to degradation. For example, Sargassum and mangroves, which serve as nurseries for juvenile fish and critical habitat for other taxa, have been hypothesised to be potential sinks for plastic pollution [76]. Long term tracking programmes can also reveal patterns of phenology or weather; the input of plastic pollution from the Ganges into marine systems is likely to be largely influenced by weather patterns, such as monsoon rains [63]. Tracking other litter items from the land could provide a better understanding of if, and when, large inputs of plastic debris into the system occur. If tracking data from vulnerable species, such as sea turtles, cetaceans and elasmobranchs, can be integrated with tracks from plastic item tags, it will help to pinpoint potential hotspot zones of interaction, such as entanglement and/or ingestion [10]. This would help guide plastic input management and clean-up efforts [10, 77].

Information gathered from movement ecology research can inform science-based policy processes [78]. To apply this method successfully for plastic pollution, individual tracking of litter items should be used in conjunction with other technologies, such as drifters, to piece together the movement and environmental processes influencing plastic debris distribution and abundance [18]. These plastic pollution tracking tools also have the ability to be used as valuable educational outreach tools for public awareness of the issue and feed into citizen science initiatives. For example, PAME’s “*Plastic in a bottle*” devices GPS transmitters send a signal every day, allowing website visitors to follow the journey of the bottles in real time on an online map [35]. They can be shown live tracked online and could be a tractable example for members of the public to understand the capacity for plastic pollution to move in riverine and marine systems. There is evidence that social marketing using tools such as these can be enablers of sustained engagement and behaviour change [79]. For example, the first tagged whale shark in the NW Atlantic @WhaleSharkRocky has 16.1K followers on Twitter [80]. Likewise, the data collected by these tags will also create leverage for stakeholders to inform policy and government regulations. Open source data collection approaches to support conservation links with one of the key objectives of the The UN Decade of Ocean Science for Sustainable Development (2021–2030) [81].

## Conclusion

Rivers connect most of the terrestrial surface of Earth to the marine environment and therefore play a critical role in the movement of plastic waste [14]. Tracking plastic litter is an opportunity to expand the use of movement ecology to aid in understanding its abundance, distribution and sources [25]. Here we have presented a low cost, open source methodology piloted in the Ganges and the Bay of Bengal to track the movement of plastic litter items in order to better understand the journey of plastic pollution in aquatic systems. The development of novel technology and the application to the global issue of plastic pollution has the potential to significantly aid in the acquisition of knowledge and be a powerful tool for stimulating social behaviour change.

## Supporting information

S1 Fig Bottle tag design and construction: a) top view showing the shape and profile of the internal bottle insert’s lid b) Side view of plastic water bottle showing the cut location c) top view showing mounting hole locations d) top down view showing placement of Arribada Horizon GPS tracker board and ARGOS R2 transmitter inside the internal enclosure e) top down view showing cable assembly positioning f) top down view showing antenna ground plane extension tabs connecting the two PCBs together g) top down view of base showing location of o-ring seal h) perspective view of base enclosure showing 6 x AA battery insert positions i) top down view showing placement of battery board in the base of the enclosure j) Side view of plastic water bottle showing internal insert positioned inside. The ARGOS antenna is not shown.
(JPG)

S2 Fig Internal enclosure: The top, front and rear view of the internal enclosure with the self-tapping screw holes visible. (JPG)

S3 Fig Orientation of batteries inside the bottles internal cavity. (JPG)
