# Correction: Message in a bottle: Open source technology to track the movement of plastic pollution

**DOI:** 10.1371/journal.pone.0269218

**Published:** 2022-05-25

**Authors:** Emily M. Duncan, Alasdair Davies, Amy Brooks, Gawsia Wahidunnessa Chowdhury, Brendan J. Godley, Jenna Jambeck, Taylor Maddalene, Imogen Napper, Sarah E. Nelms, Craig Rackstraw, Heather Koldewey

In the Funding statement, the source of salary for Gawsia Wahidunnessa Chowdhury is listed incorrectly. The correct funder is “University of Dhaka, Bangladesh.”
