# Supplementary figures and images for: Message in a bottle: Open source technology to track the movement of plastic pollution
Source: PLoS One. 2020 Dec 2;15(12):e0242459. doi: 10.1371/journal.pone.0242459 (PMC7710111; doi:10.1371/journal.pone.0242459)

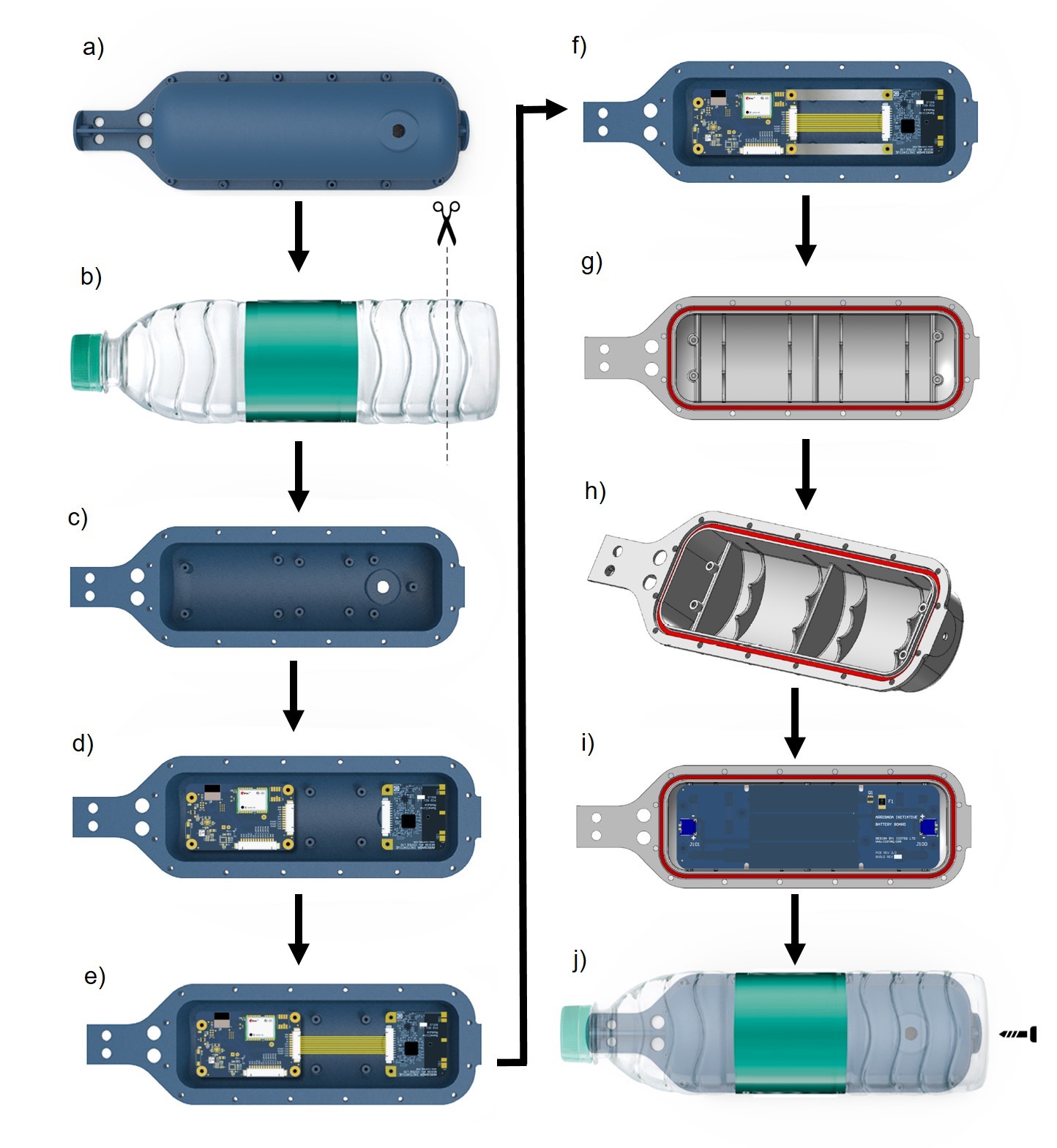

Supplement: S1 Fig — The ARGOS antenna is not shown. (JPG) [file pone.0242459.s001.jpg]

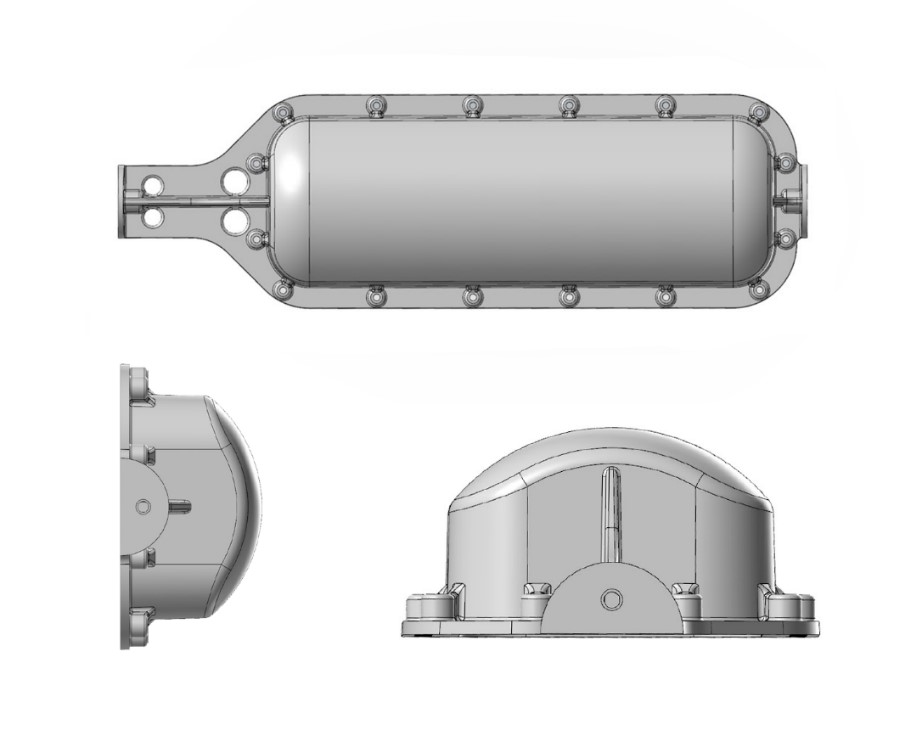

Supplement: S2 Fig — (JPG) [file pone.0242459.s002.jpg]

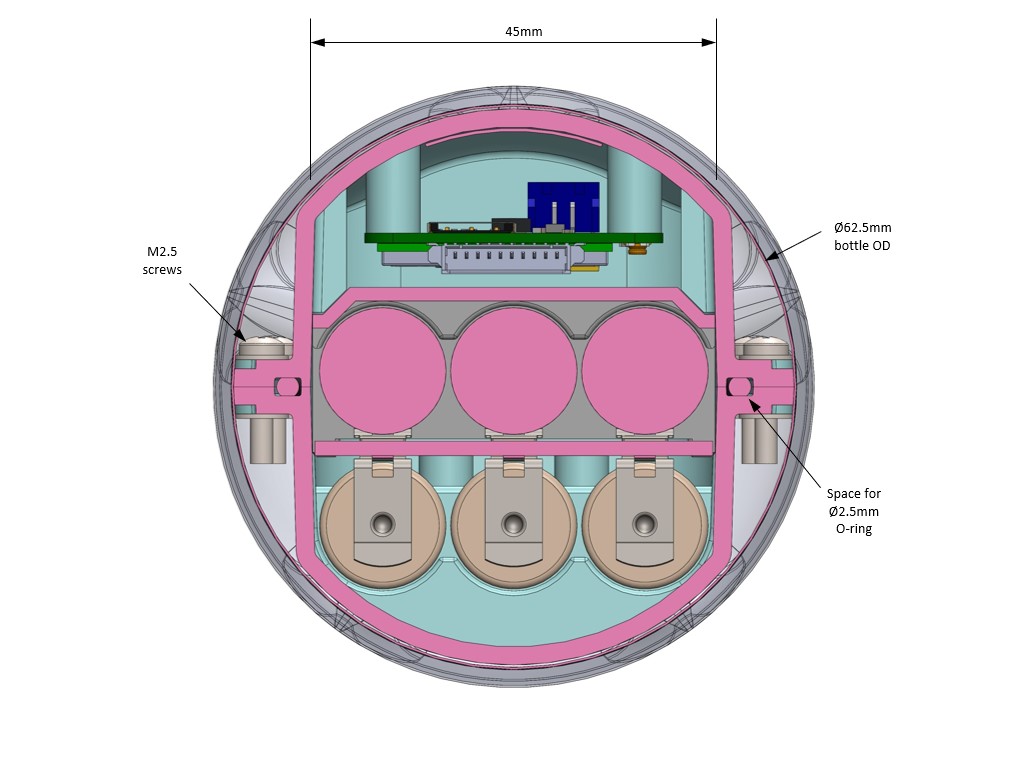

Supplement: S3 Fig — (JPG) [file pone.0242459.s003.jpg]
